# Application of the improved simple bedside method for emergency temporary pacemaker implantation suitable for primary hospitals

**DOI:** 10.1038/s41598-021-96338-z

**Published:** 2021-08-19

**Authors:** Changqing Zhong, Shanjun Mao, Jieli Guang, Yi Zhang

**Affiliations:** 1 Department of Cardiovascular Medicine, Hunan Provincial People’s Hospital (First Affiliated Hospital of Hunan Normal University), Changsha, Hunan China; 2 Department of Cardiovascular Medicine, First Affiliated Hospital of Hunan Normal University, Changsha, Hunan China; 3 Department of Statistics, College of Finance and Statistics, Hunan University, Room 228, Unit 2, Red Building, Finance Campus, Changsha, Hunan China; 4 Department of Internal Medicine, Yunnan Normal University Hospital, Kunming, Yunnan China

**Keywords:** Cardiology, Medical research

## Abstract

The purpose of the research was to evaluate the safety and effectiveness of the X-ray-free improved simple bedside method for emergency temporary pacemaker implantation as well as the practicability of the method in primary hospitals. Patients [including those suffering from sick sinus syndrome and third-degree and advanced atrioventricular blockage (AVB)] who needed emergency temporary pacemaker implantation from July 2017 to August 2020 in Hunan Provincial People’s Hospital were selected. They were stochastically divided into a research group (95 cases) treated with the improved simple bedside method and a control group (95 cases) with X-ray guidance. The ordinary bipolar electrodes were used in both groups. On this condition, the operation duration, the first-attempt success rate of electrodes, pacing threshold, success rate of the operation, the rate of electrode displacement, and complications in the two groups were separately calculated. The comparison results of the research group with the control group are shown as follows: operation time [(18 ± 5.91) min vs. (43 ± 2.99) min, *P* < 0.05], the first-attempt success rate of the electrode (97% vs. 98%, *P* > 0.05), pacing threshold [(0.97 ± 0.35) vs. (0.97 ± 0.32) V, *P* > 0.05], success rate of the operation (98.9% vs. 100%, *P* > 0.05), the rate of electrode displacement (8.4% vs. 7.3%, *P* > 0.05) and complications (3.2% vs. 2.1%, *P* > 0.05). The emergency temporary pacemaker implantation based on the improved simple bedside method is as safe and effective as the surgical method under X-ray guidance, and the operation is simpler and easier to learn and requires a shorter operating time, therefore, it is more suitable for use in emergency and primary hospitals.

## Introduction

Emergency temporary pacemaker implantation is first aid measure for patients with severe bradyarrhythmia and cardiac arrest^1–3^. At present, there are three main clinical methods of temporary pacemaker implantation: X-ray guidance, bedside balloon-tipped floating catheter, and ordinary bipolar electrode placement methods. Although temporary pacemaker implantation with the ordinary bipolar electrode under X-ray guidance is both safe and effective, the surgery needs to be conducted under X-ray guidance. On the one hand, this takes precious time when rescuing patients^4–6^; on the other hand, patients and surgeons are exposed to X-rays. In addition, onerous requirements such as the demand for a C-arm device and a specialized catheterization room are not conducive to application of the procedure in the emergency departments of many primary hospitals not meeting these conditions^7^. The bedside balloon-tipped floating catheter implantation is another rapid and effective first aid method^8,9^. However, few balloon floating electrodes are used in many primary hospitals in China. One reason is that the balloon-tipped floating catheter is more expensive than that of the ordinary bipolar electrode. As a result, the increase of expenditure on the part of the patients and medical insurance providers actually restricts the popularization and application of the balloon-tipped floating catheter in the primary hospitals in developing countries. Hence, emergency temporary pacemaker implantation at the bedside was modified by applying the ordinary bipolar electrode without X-ray guidance, expecting that the implantation method can be implemented by doctors in primary hospitals under simple conditions. A comparative study was conducted on the simple bedside method and X-ray guidance method based on patients who needed emergency temporary pacemaker implantation in Hunan Provincial People’s Hospital in the most recent 3-year period (July 2017 to August 2020). On this basis, the safety and effectiveness of the improved X-ray-free simple bedside method for emergency temporary pacemaker implantation as well as its practicability in primary hospitals were discussed.

## Data and methods

**Figure 1:**
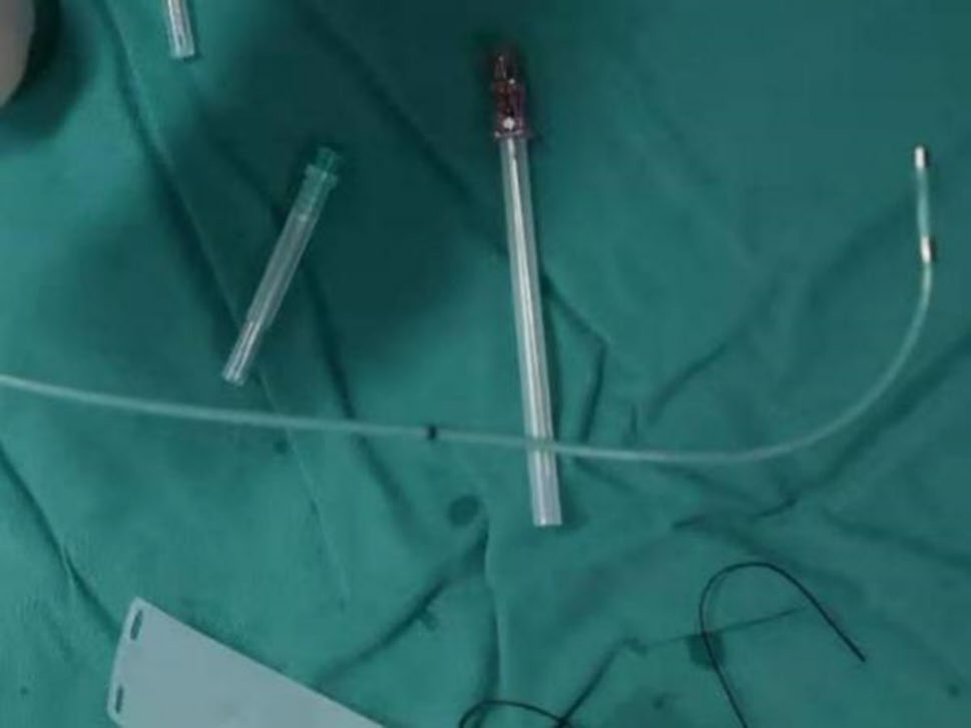
The bipolar electrode shaped as a quarter bend.

**Figure 2:**
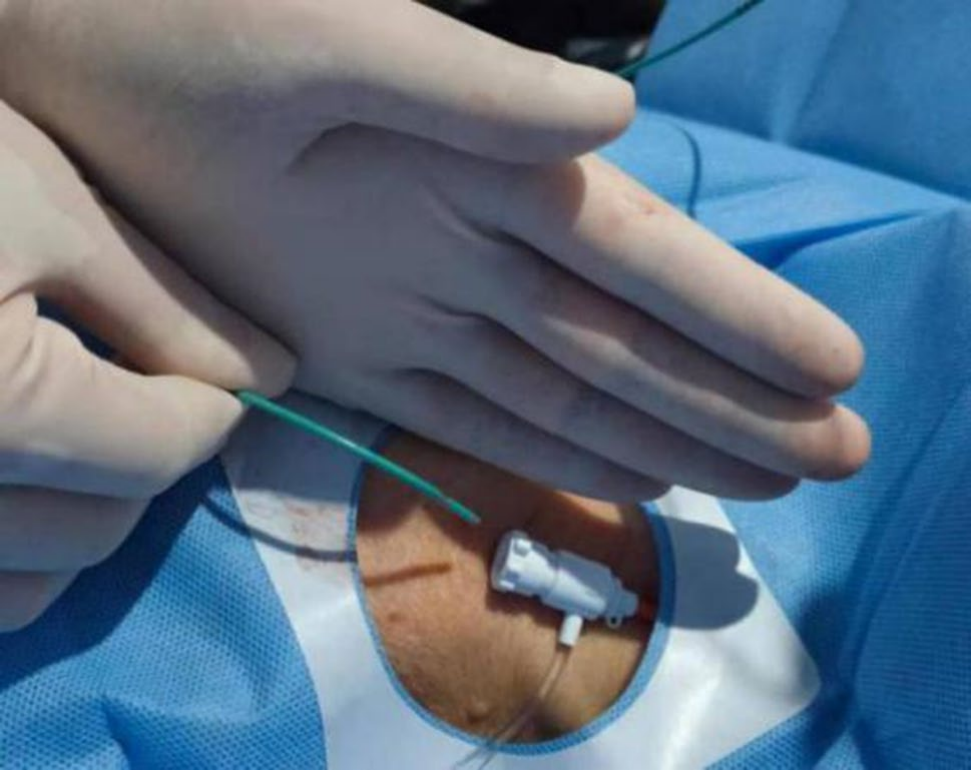
Forming an angle of 30° between the electrode and the sagittal section.

1. Research objects: The patients who needed emergency temporary pacemaker implantation from July 2017 to August 2020 in Hunan Provincial People’s Hospital were screened according to the following inclusion criteria: (1) showing bradycardia associated symptoms (such as amaurosis and syncope); (2) electrocardiograph (ECG) or dynamic ECG hints sinus arrest or sino-atrial block, with the average heart rate lower than 40 bpm, and third-degree and advanced atrioventricular blockage (AVB). The patients who did not sign the informed consent proforma and that did not cooperate were excluded. In addition, a statement is worth mentioning. All experiments were performed in accordance with relevant guidelines and regulations, and all the data were approved by Hunan Provincial People's Hospital in China.
2. Research methods: The research objects were stochastically divided into a research group (95 cases) treated with the improved simple bedside method and a control group (95 cases) treated under X-ray guidance. The patients (102 males and 88 females) of ages of 15 to 87 years had a mean average age of 62 ± 10.45 years. Surgery in the two groups was completed by the primary hospitals doctors under electrophysiologists or interventional cardiologists guidance by puncturing the right internal jugular vein. After the patients and their families signed the informed consent proforma, the bedside ECG monitoring was conducted on the patients in the research group. The patients in the supine position were locally anaesthetized with lidocaine after routine disinfection and draping. After successful puncture, the guide wire and 7F sheath were placed into the patients. The ordinary bipolar electrodes produced by SJM Corporation were bent to have a quarter bend in vitro (Fig. 1); afterwards, the top of the electrode was placed towards the lower left, forming an angle of 30° with the sagittal section (Fig. 2), and entered the right ventricle along the vessel sheath, with the insertion length being (height (cm) + 180)/10. If new ventricular premature beat (VPB) or short-run ventricular tachycardia was found through ECG monitoring, the first attempt was deemed successful. In this case, it was feasible to make a connection to the temporary pacemaker transmitter to test the pacing effect. If the electrode was not successfully inserted, the electrode was pulled out and the process repeated. As for the control group, it was necessary to send the patients to the catheter room. Similarly, the operation was conducted by puncturing the right internal jugular vein after routine ECG monitoring, disinfection, draping and local anesthesia. The guide wire and the vessel sheath were placed under X-ray guidance. Subsequently, the ordinary bipolar electrode (produced by SJM Corporation) was placed into the right ventricle under X-ray guidance along the vessel sheath. In terms of the insertion length, the electrode tip needed to be precisely located in the low septum at the ventricular apex under fluoroscopy. Afterwards, it was essential that it was connected to the temporary pacemaker transmitter. The pacing threshold was tested in the two groups. The lowest pacing voltage, under which the ventricle capture failed during pacing by gradually decreasing the pacing output, was determined as the pacing threshold. According to the threshold, the pacing voltage was set within the range of 3.5 to 5.0 V, with a sensing resolution of 2.0 mV and a pacing frequency of 1 Hz. Finally, whether the electrode reached the target position was judged according to the bedside ECG monitoring, that is, a downward V1 main wave, downward main waves in leads II, III, and AVF (right ventricular apex), a negative R-wave in the lead AVR, and positive waves in leads II, III, and AVF, which strongly hinted that the top of the electrode was located in the right ventricular outflow tract in the high septum. The RBBB graph indicates that the electrode was in the coronary sinus or unexpectedly reached the left ventricle^10^. All of the above research plans were approved by the Medical Ethics Committee of Hunan Provincial People's Hospital / the First Affiliated Hospital of Hunan Normal University (2017.No.26).
3. Observation indices: (1) Operation time for the two groups of cases: the duration from signing the informed consent proforma to connection of the temporary pacing electrode with the pulse generator, showing a favorable pacing effect; (2) the first-attempt success rate of the electrode: the success rate of placing the electrode in the low septum at the right ventricular apex for the first time during operation; (3) pacing threshold: the lowest pacing voltage measured during the operation; (4) the success rate of the operation: it is successful if the operation is completed and the pacing and sensing functions are favorable; (5) complications: mainly including hematoma, pneumothorax, cardiac perforation, and pericardial effusino during and for 5 days after the operation; (6) the rate of electrode displacement at 5 days after the operation: the condition that requires replacement of the pacing electrode due to pacing and sensing abnormalities caused by the post-operative electrode displacement.
4. Statistical treatment: The data were input by applying EpiData 3.1 and statistical analysis was performed on the input data based on SPSS22.0. The ages of patients, operation time, and pacing threshold are considered as measurement data, which are expressed by using $\overline{x} \pm s$; moreover, an inter-group comparison was conducted by utilizing a *t-*test. The gender, the first-attempt success rate of the electrode implantation, complications, the rate of incidence of electrode displacement, and the success rate of the operation are taken as the enumerative data, which are expressed in percentage terms. The inter-group comparison was performed by using the *χ*^2^ test wherein *P* < 0.05 denotes a difference that is statistically significant.

## Results

1. Comparison of general clinical data of two groups: there were 95 patients in the research group, including 52 males (54.74%, 52/95) and 43 females (45.26%, 43/95), with the average age of 61.00 ± 12.62 years old; the control group covered 95 patients, including 50 males (52.63%, 50/95) and 45 females (47.37%, 45/95), with the average age of 63.00 ± 10.86 years old. By comparing the two groups of patients in terms of the gender and age, the differences showed no statistical significance (*χ 2* = 0.013, *P* > 0.05; *t* = 0.115, *P* > 0.05). It can be found that there was no significant difference in the two groups in terms of gender and age (*P* > 0.05).
2. Comparison of the observation indices in two groups: the operation was successfully completed in 94 cases in the research group while failed in the case of one patient. In that patient, the electrode was successfully placed under X-ray guidance in the catheter room. The operation was successfully completed in 95 cases in the control group. In the research group, two cases were subjected to slight hematoma at the puncture position and one patient experienced slight pericardial effusion; slight hematoma was found at the puncture positions in two cases in the control group. The differences in the first-attempt success rate of the electrode, pacing threshold, the rate of electrode displacement, success rate of the operation, and complications in the two groups showed no statistical significance (*P* > 0.05); however, a significant difference (*P* < 0.05) was present in the operating time of the two groups (Table 1). Moreover, a power analysis was conducted to show the reliabilities of the above tests. The power was fixed at 0.9 and the corresponding significance levels of these tests were displayed in Table 1. We could find that the test about operation time had a low significance level which means that it has a very low probability of making both types of errors, and other tests had high significance levels which mean that there are high probabilities of rejecting these null hypotheses when they are true, however, the outcomes obtained by the data just do not reject these null hypotheses, thus increasing the authenticity of the outcomes. Overall, the results indicated that the above conclusions were reliable, that was, the new methods did not cause any loss in first-attempt success, pacing threshold, success rate, electrode displacement and complication, but it could reduce the operation time significantly.

**Table 1:** Comparison of observation indices.

Groups	Number of cases	Operation time (min) $\overline{x} \pm s$	First-attempt success rate of the electrode (%)	Pacing threshold $\overline{x} \pm s$	Success rate of the operation (%)	Rate of the electrode displacement (%)	Proportion of complications (%)
Research group	95	18 ± 5.91	97%	0.97 ± 0.35	98.9%	8.4%	3.2%
Control group	95	43 ± 2.99	98%	0.97 ± 0.32	100%	7.3%	2.1%
*χ*^2^/*T* value		*t* = 28.592	*χ*^2^ = 0.205	*t* = 0.000	*χ*^2^ = 1.003	*χ*^2^ = 0.072	*χ*^2^ = 0.117
*P* value		< 0.05	> 0.05	> 0.05	> 0.05	> 0.05	> 0.05
Significance level with power = 0.9		0.01%	89.0%	90.0%	72.6%	89.6%	88.8%

## Discussion

At present, many more bradycardia patients are subjected to emergency medical treatment due to the occurrence of amaurosis, syncope, and Adams-Stokes syndrome (such as sick sinus syndrome and third-degree AVB (AVB-III)) in many primary hospitals at county level or below in China. It requires the doctors in these hospitals to master the operative technology of rapidly implanting an emergency temporary pacemaker, rather than transferring the patients to a superior hospital with concomitant higher risk^11,12^. Previously, primary hospitals rarely conduct such first aid treatment because of limitations imposed by equipment conditions and the operator skill-level required. The comparison of results between the research and control groups show that no statistical difference is found in the pacing threshold, the first-attempt success rate of the electrode, success rate of the operation, the rate of electrode displacement, and other complications. This suggests that emergency temporary pacemaker implantation based on the simple bedside method without X-ray is as safe and effective as the operation conducted under X-ray guidance in the catheterization room. In the research group, the temporary pacing electrode failed to be implanted into a patient after repeated attempts based on the simple bedside method; finally, it was successfully implanted under X-ray guidance in the catheterization room. While, it was validated by the post-operation ultrasonic cardiogram (UCG) that the patient had an Ebstein anomaly: the right atrium of the patient was dilated (reaching 51 mm in diameter), showing an atrial septal defect, dilated coronary sinus (DCS), and downward displacement of tricuspid valve. Therefore, it was difficult to get the ordinary bipolar electrode to cross the tricuspid valve to reach the target position in the right ventricle by using the simple bedside method; however, congenital heart disease (CHD) with multiple abnormalities is uncommon, thus, in this case, emergency temporary pacemaker implantation is challenging at the bedside without X-ray guidance and it is possible that repeated attempts may still fail. It increases the risk of cardiac perforation and cardiac tamponade. Under these conditions, it is necessary to transfer the patient to a hospital with a specialized catheterization room to perform pacing electrode implantation under X-ray guidance as early as possible. Certainly, it is beneficial if the surgeon can decide how to act in advance on the basis of the UCG result before the operation. The emergency temporary pacemaker implantation was successful in the other 94 patients in the research group based on the simple bedside method without X-ray guidance. Among the 94 patients, the electrode was successfully implanted in the first attempt in 97% of patients; as for the other 3% of patients, the electrode was also successfully implanted at the second attempt by repeating the same procedure after pulling out the electrode. The possible reason why the electrode was not accurately implanted at first attempt in a small number of patients was that the surgeon failed to make the lower left-hand end of the top of the electrode form an initial included angle of 30° with the sagittal section.

**Figure 3:**
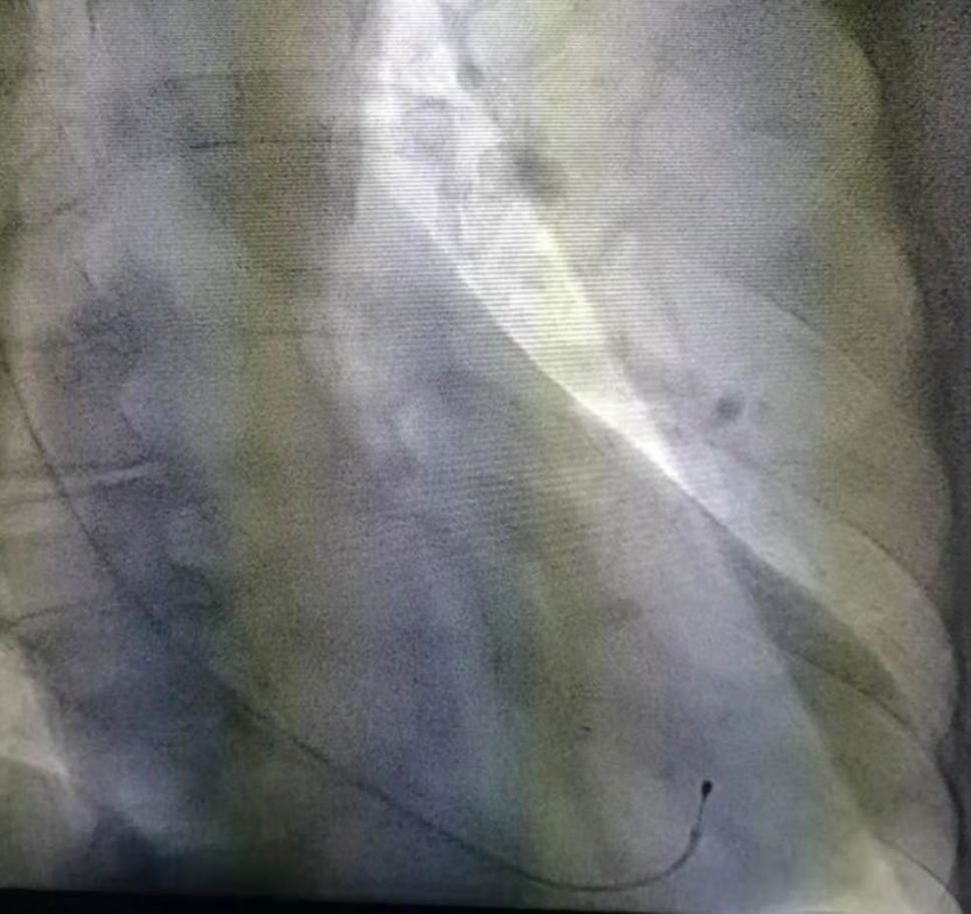
An electrode with its tip located in the low septum at the right ventricular apex.

In terms of complications, three patients in the research group developed complications, including slight hematoma at the puncture positions in two patients along the internal jugular vein and slight pericardial effusion in one patient; two patients in the control group developed slight hematoma. There was no statistical difference between the two groups. The occurrence of hematoma was attributed to the fact that the doctors in primary hospitals had only recently learned the technique required for internal jugular vein puncture and they may have punctured the internal carotid artery during the operation. After timeous application of pressing for about 5 min, the hematoma was naturally absorbed and faded 1 to 2 days after the operation, without discomfort. With the increase in the number of cases subjected to puncture, hematoma occurred less often and no pneumothorax was observed in the two groups. This indicated that, under emergency bedside conditions, it was safe and reliable for relatively inexperienced primary doctor to implant temporary pacemakers by preferentially selecting internal jugular vein puncture^2,13–15^. The reason for this is that the internal jugular vein is directly connected to the superior vena cava, showing a large lumen diameter and short travel path. In addition, there is no bifurcation and angulation while there is obvious surface landmark, which makes the puncture convenient, contributing to a high rate of electrode implantation and a lower probability of occurrence of pneumothorax compared to the risk of puncturing the subclavian vein, Other complications are also low^16,17^. In the research group, slight pericardial effusion was found while no cardiac tamponade appeared in the patient 3 days after the operation. The UCG result suggested that both the right atrial and right ventricular diameters of the patient were much smaller than those in a normal subject. Fluoroscopy showed that the shaping tension of the electrode was larger, and the electrode tip did not penetrate the pericardial cavity. By retracting the electrode by about 1 cm, the pericardial effusion was naturally absorbed after 2 days, therefore, the improved calculation formula ((height (cm) + 180)/10) for the insertion length of the electrode was applicable to the majority of people. After the operation, X-ray examination validated that the electrodes were all located in the low septum at the right ventricular apex (Fig. 3); however, it was necessary to increase or reduce the insertion length by between1 to 2 cm in patients whose right atrium and right ventricle are significantly dilated or contracted. In this way, the incidence of pericardial effusion and pacing abnormality can be reduced. Although there is no statistical difference between the incidence of complications in the normal electrode installation without the X-guided bed and in the X-guided installation. But some studies have been reported that the complications of the bedside balloon floating electrode installation were low^18^, because the tip of the latter electrode was very soft. Therefore, the beside balloon floating electrode installation, if followed by our improved simple approach, may be an appropriate approach that is likely to reduce complications and dislocation rates, which we will consider in our future studies. Moreover, complications and displacement rate are not only related to technique methods and electrode materials, but also related to the training of operators^19,20^.

The operation time in the research group is much shorter than that in the control group. The reason for this is that the operation (among those in the control group) was conducted in a specialized catheterization room under X-ray guidance, showing evidence of increasing uncontrollable factors and links: as a result, the operation was prolonged, therefore, for those emergency patients who aim to exploit precious rescue time to best effect, there is no doubt that emergency temporary pacemaker implantation based on the simple bedside method shows great superiority, especially for those subjected to initial surgical treatment in the emergency department of primary hospitals.

Some cases in the control group show that the electrode is more likely to be poorly placed when the insertion length in the heart cavity is too large due to the natural deflection of the heart cavity, thus leading to poor pacing. Generally, relative to the low septum at the right ventricular apex, the temporary pacing electrode at the right ventricular outflow tract in high and middle septum is more likely to be badly placed, thus triggering a pacing and sensing abnormality. Right ventricular high and mid-septal pacing is closer to the ventricular activation sequence and ventricular synchrony in the natural physiological state^21–23^. Nevertheless, it is most important for the patients subjected to the temporary pacemaker implantation to ensure safe and stable pacing. Right ventricular apex pacing is likely to induce ventricular mechanical asynchrony and increase mitral and tricuspid regurgitation, thus influencing ventricular systolic function owing to the different electrical activation sequence from the physiological activation sequence^24^. Despite this, surgeons prefer right ventricular apex pacing as the temporary pacemaker is generally used as a first aid measure in the transition period, with an application time of only 3 to 5 days, which does not trigger the rapid degradation of cardiac function.

**Figure 4:**
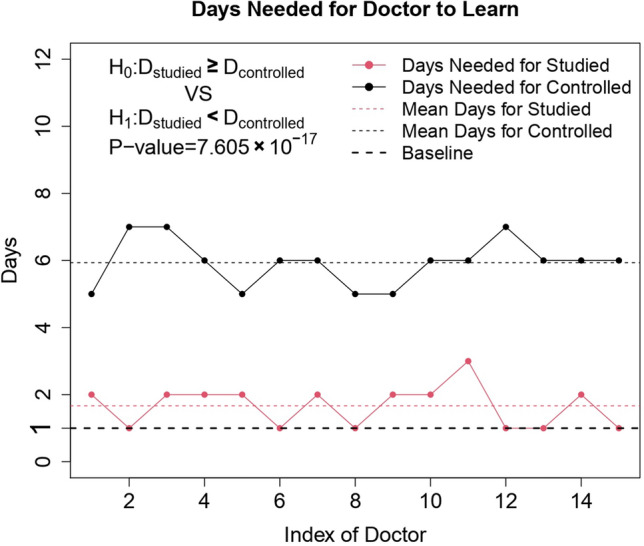
Training curves of primary care doctors.

The operations were completed by primary care doctors trained in the hospital. Among 15 trained primary care doctors in the research group, six, eight, and one of them were trained by separately performing one, two, and three operations. They all mastered all operation processes and could independently complete the subsequent surgical procedures in all cases. Among 15 trained primary care doctors in the control group, four, eight, and three doctors were trained by separately conducting five, six, and seven operations. Afterwards, they were able to complete the surgical procedures of the subsequent cases independently. The results showed that the emergency temporary pacemaker implantation based on the improved simple bedside method requires only simple and convenient operational processes and entails a short learning curve, making it suitable for use in emergency and primary hospitals (Fig. 4). It would also be helpful to change the status that many centers rely on specially trained electrophysiologists or interventional cardiologists to complete the operation^25,26^.

The emphases and procedures of the simple bedside method are described as follows: (1) preferentially placing the catheter along the right internal jugular vein; (2) shaping the ordinary bipolar electrode into a quarter bend in vitro; (3) when inserting the electrode, the top points to the lower left to form an included angle of 30° with the sagittal section, leaving the overall direction unchanged; (4) the insertion length is calculated as (height (cm) + 180)/10; moreover, it is necessary to increase or reduce the insertion length by 1 to 2 cm in patients whose right atrium and right ventricle are significantly dilated or contracted according to whether VPB caused by electrode contact occurs (as evinced by ECG monitoring).

Above all, the research results revealed that emergency temporary pacemaker implantation based on the simple bedside method without X-ray guidance is safe, efficient, and practical with the following advantages: (1) Installation electrode and adjustment electrode position require no X-ray guidance, and no X-ray exposure from both doctors and patients; (2) it is not necessary to conduct complex learning and training for identification of the catheter insertion site according to ECG, resulting in a short learning curve among surgeons. The method is more applicable to popularization and application in primary hospitals; (3) as for the critical patients who should not be moved, it is feasible to implant a temporary pacemaker at the bedside, thus winning more rescue time: however, for a small number of patients suffering from CHD with multiple structural abnormalities, such as large atrial and ventricular septal defects and downward displacement of tricuspid valve, it is more appropriate to employ the temporary pacemaker implantation under X-ray guidance. It is necessary to select other puncture paths in some cases with a blocked right internal jugular vein.

This study has limitations. The balloon floating electrodes is not considered in the study based on the reason mentioned in the discussion section. If the balloon float electrodes is adopted by following our improvement method, it is expected to further reduce complications and dislocation rates. This is well worth exploring in future research, and we’ll take this into serious consideration in our future experiments.

## Data Availability

All data generated or analyzed during this study are included in this published article.
